# Conjugation of Natural Triterpenic Acids with Delocalized Lipophilic Cations: Selective Targeting Cancer Cell Mitochondria

**DOI:** 10.3390/jpm11060470

**Published:** 2021-05-25

**Authors:** Anna Yu. Spivak, Darya A. Nedopekina, Rinat R. Gubaidullin, Mikhail V. Dubinin, Konstantin N. Belosludtsev

**Affiliations:** 1 Organic Synthesis Laboratory, Institute of Petrochemistry and Catalysis, Russian Academy of Sciences, 450075 Ufa, Russia; spivak.ink@gmail.com (A.Y.S.); rawbe2007@mail.ru (D.A.N.); rinatg83@mail.ru (R.R.G.); 2 Department of Biochemistry, Cell Biology and Microbiology, Mari State University, 424001 Yoshkar-Ola, Russia; dubinin1989@gmail.com; 3 Laboratory of Mitochondrial Transport, Institute of Theoretical and Experimental Biophysics, Russian Academy of Sciences, 142290 Moscow, Russia

**Keywords:** mitochondria, mitochondrial targeting, delocalized lipophilic cations, anti-cancer agents, mitocans, triterpenic acids

## Abstract

Currently, a new line of research on mitochondria-targeted anticancer drugs is actively developing in the field of biomedicine and medicinal chemistry. The distinguishing features of this universal target for anticancer agents include presence of mitochondria in the overwhelming majority, if not all types of transformed cells, crucial importance of these cytoplasmic organelles in energy production, regulation of cell death pathways, as well as generation of reactive oxygen species and maintenance of calcium homeostasis. Hence, mitochondriotropic anticancer mitocan agents, acting through mitochondrial destabilization, have good prospects in cancer therapy. Available natural pentacyclic triterpenoids are considered promising scaffolds for development of new mitochondria-targeted anticancer agents. These secondary metabolites affect the mitochondria of tumor cells and initiate formation of reactive oxygen species. The present paper focuses on the latest research outcomes of synthesis and study of cytotoxic activity of conjugates of pentacyclic triterpenoids with some mitochondria-targeted cationic lipophilic molecules and highlights the advantages of applying them as novel mitocan agents compared to their prototype natural triterpenic acids.

## 1. Introduction

Despite the considerable progress over the past few decades in the treatment of cancer, it remains the main threat to human health and the second leading cause of death after cardiovascular disease [1]. The transformation of normal cells into a malignant form involves multiple genetic and metabolic changes resulting in hyperproliferation, apoptosis resistance and evasion of the host immune response. Most of the current antitumor chemotherapy drugs, including natural compounds of plant origin (vinca alkaloids, taxanes, podophyllotoxins, captothecins), target the genome or mechanism of division of tumor cells (intercalation and DNA repair, microtubular destruction). Still, practically all existing anticancer cytostatic drugs have severe side effects on account of the low selectivity of the antiproliferative action and develop multiple drug resistance of tumors to chemotherapy. The next generation targeted anticancer drugs have high selectivity of action and low systemic toxicity, but they do not actually address the problem of tumor resistance to therapy arising from high heterogeneity and mutation of the genetic apparatus of cancer cells [2]. Indeed, the experiments demonstrate that even in the same patient, tumor cells differ in expressed genes and existing mutations within the same tumor [3,4]. Moreover, in heterogeneous solid tumors, anoxic and hypoxic zones are formed, within which a special clinically significant cell population, that is cancer stem cells (CSC), develops. These cells adapt well to low oxygen concentrations and exhibit high capacity to repopulate tumors [5].

Thus, research and development of new synthetically available antitumor agents, a fundamentally different mechanism of action against resistant tumors, of a high therapeutic selectivity index and acceptable pharmacokinetic parameters, remain an urgent priority. An example of such developments is the creation of small molecules known as mitocans (acronym derived from the terms mitochondria and cancer). According to modern concepts, these compounds have high anti-cancer potential [6,7,8]. A number of mitocans have shown efficacy in selective cancer cell killing in pre-clinical and early clinical testing [9]. Mitocans destabilize the mitochondria of some tumor cells and induce mitochondria-dependent cell death. The selective anti-cancer effect of mitocans is associated with some functional and structural differences between the mitochondria of healthy and cancer cells [9,10].

There are several approaches to create mitocans, including conjugation of a biologically active compound with mitochondria-targeting “vehicles”. Various positively charged molecules (lipophilic penetrating cations, cationic peptides, etc.) are considered as a component that improves mitocan uptake by mitochondria. This is due to the high membrane potential (negative inside) of the inner mitochondrial membrane. A large number of compounds are considered as bioactive components. There are several mechanisms (for example, ROS overproduction, inhibition of respiratory chain complexes, etc.) that can be used to destabilize mitochondria and kill cancer cells.

In the past few years, available natural pentacyclic triterpenoids such as betulin, betulinic, ursolic, oleanolic and glycyrrhetic acids have been studied as promising scaffolds for development of new mitochondria-targeted anticancer agents. The antitumor effect of native triterpenic acids, established in vitro against various tumor cell lines (melanoma, adenocarcinoma, neuroblastoma, medulloblastoma and glioblastoma), are complemented with low systemic toxicity [11,12,13,14,15,16,17]. These secondary metabolites affect the mitochondria of tumor cells, initiating reactive oxygen species overproduction [18].

One could note that many efforts have been focused on targeted delivery of the terpenoids (for example betulin and betulinic acid) to cancer cells via encapsulation in nanoparticles. Various nano/microcarriers such as liposomes and carbon nanotubes loaded with betulin and betulinic acid have been used to develop new drugs [19]. In addition, there are reports of the use of a folate ligand to target betulinic acid to leukemic cells [20]. A polymer nano-carrier was also synthesized based on self-assembly of betulinic acid in the presence of PEG, and the carrier was modified with folic acid. In this paper, we refer to the advantages of applying conjugates of pentacyclic triterpenoids with some mitochondria-targeted cationic lipophilic molecules as potential mitocan agents compared to their prototype natural triterpenic acids and their nano-encapsulated forms.

## 2. Metabolic and Mitochondrial Changes in Cancer Cells

Tumor cells are known to exhibit substantial metabolic reprogramming. Almost a century ago, Otto Warburg suggested that tumor cells show a shift in energy metabolism from oxidative phosphorylation in mitochondria to aerobic glycolysis [21]. However, in recent years, the “Warburg effect” has been revised and thoroughly studied using advanced approaches (transcriptomics, proteomics, metabolomics, etc.). It was found that tumor cells show an increase in glucose consumption, which is facilitated by the rearrangement of the glycolytic pathway, including the over-expression of pyruvate kinase M2 (PKM2), leading to a backup of upstream glycolytic phospho-intermediates; glucose-6-phosphate dehydrogenase, promoting the generation of pentose phosphates for ribonucleotide synthesis and NADPH production; dihydroxyacetone-phosphatase, providing biosynthesis of cell membrane components; phosphoglycerate dehydrogenase, supplying cancer cells with essential amino acids serine and glycine [22,23]. These and other examples suggest that the enhancement of aerobic glycolysis is used to create the building blocks of macromolecules for biosynthetic processes that are critical for accelerated tumor growth [24], rather than for energy production, as originally thought. Subsequently, inhibitors of glycolysis enzymes have been used in the treatment of certain tumors [25].

Mitochondria are one of the key objects of research in various fields of biomedicine, including cancer therapy. These organelles are the main source of energy in the form of ATP in eukaryotic cells and are also involved in the generation of reactive oxygen species, the production of intermediate metabolites, regulation of Ca^2+^ homeostasis and thermogenesis. The functions of mitochondria in many processes of cellular physiology/pathophysiology, including cell survival and proliferation, cell signaling, neoplasia and cell death, have been detailed [26,27,28,29,30,31,32,33]. Mitochondria are known to contribute to malignant transformation and tumor progression by increasing the plasticity of cancer cells and controlling some of the mechanisms necessary for their work.

Some tumors have been shown to be highly dependent on mitochondrial oxidative phosphorylation [34,35,36] and biosynthetic processes [37,38]. In addition, an increased dependence of tumor cells on OXPHOS was observed in the later stages of the disease [22]. Moreover, one should note that the use of complex I inhibitor IACS-010759 suppressed proliferation and induced apoptosis in models of brain cancer and acute myeloid leukemia (AML) reliant on OXPHOS [39]. On the other hand, some works noted the fact that the mitochondria of cancer cells show an increased membrane potential [40]. Indeed, according to various sources, the transmembrane potential of normally mitochondria carry around 150–180 mV [41,42] and can reach 210 mV in some cancer cells (Neu4145 cancer cells) [43]. It has been suggested that this may be due to the fact that the inner mitochondrial membrane of transformed cells is marked by an increase in cholesterol and cardiolipin, contributing to a decreasing intensity of passive proton leak through the membrane [44,45]. One could speculate that mitocans synthesized on the basis of penetrating cations will accumulate mainly in the mitochondria of cancer cells. The difference (60 mV) for ΔΨmito between solid tumor cells and normal cells can bring about a 10-fold increase in the selective targeting of the cationic compound in the mitochondria of cancer cells [9,46,47,48]. However, it is difficult to say how true this statement is. Indeed, the cations used can also accumulate in the mitochondria of normal cells, which requires careful selection of the configuration and concentration of the therapeutic agent for a targeted effect on tumor cells. Moreover, the existing methods for measuring the mitochondrial potential do not allow us to unambiguously assert an increase in the membrane potential in the mitochondria of cancer cells compared to healthy ones. In this case, it is impossible to exclude other factors contributing to high retention of cations in the mitochondria of tumor cells.

Cancer cells show dysregulation of mitochondrial metabolism mediated by mutations in the nuclear or mitochondrial genomes. This can lead to dysfunction of proteins of the Krebs cycle and OXPHOS and the accumulation of various metabolites (oncometabolites) in mitochondria (and then in the cytoplasm), which can modulate the metabolic flux through the direct regulation of gene expression or other metabolic pathways [10]. In particular loss-of-function mutation of succinate dehydrogenase has been associated with several types of cancer [49]. Accumulation of succinate leads to suppression of prolyl hydroxylase, stabilization of HIF1a and stimulation of the glycolytic pathway. In this regard, this signaling function of mitochondria is strictly related to the modulation of cancer cell metabolism, which supports many cancer-related functions, such as cell proliferation, migration and resistance to death [10].

Oncogene activation, tumor suppressor loss, cancer-inducing mutations in TCA cycle enzymes and hypoxia lead to the production of abnormal mitochondrial ROS levels [50]. In this case, a high level of expression of antioxidant proteins is also observed. The mitochondrial ROS is believed to activate different signaling pathways towards protumoral metabolic reprogramming. At the same time, targeting mitochondrial ROS and antioxidant systems could be beneficial as anticancer therapy. This is due to the fact that the consequence of increased ROS production is a lower threshold of sensitivity to ROS-induced apoptosis of cancer cells [51].

Thus, in cancer cells, mitochondria undergo structural and functional rearrangements that affect the work of TCA, the mitochondrial respiratory chain, Ca^2+^ homeostasis, production of ROS, oncoproteins and oncometabolites, etc. Functional mitochondria are essential for tumor growth [52] mostly due to their biosynthetic role rather than their proenergetic features [53]. For a more detailed analysis of changes in the functioning of mitochondria in cancer cells, we suggest referring to the following excellent reviews [10,54].

## 3. Delocalized Lipophilic Cations (DLCs) for Mitochondria-Targeted Drug Delivery

**Figure 1:**
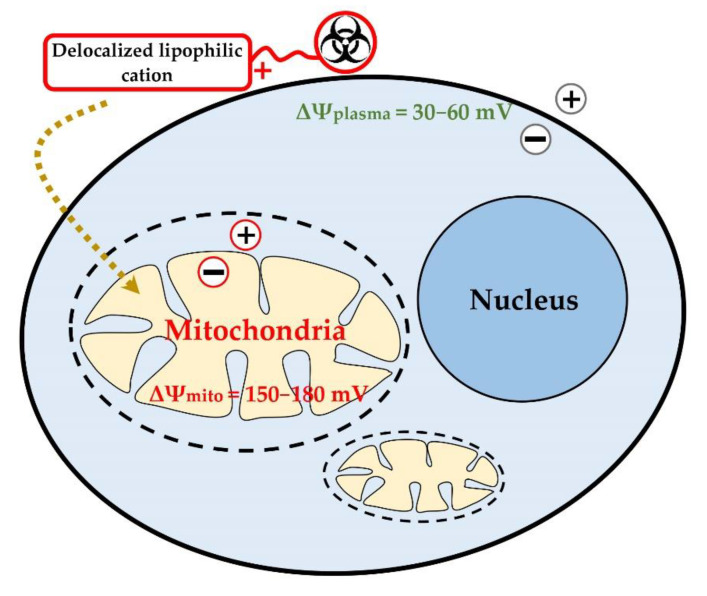
Cellular uptake of DLCs-based compounds driven by cell membrane potential and mitochondrial membrane potential.

At present, various strategies for targeted delivery of biologically active molecules and drugs to mitochondria are under study, including conjugation of biologically active compounds with cations of lipophilic molecules of low molecular weight, which can accumulate inside mitochondria [9,46,47,48,55,56,57,58]. These small molecules easily penetrate into mitochondria due to the greater value of the transmembrane potential compared to the potential of the cell membrane (ΔΨmito = 150–180 mV, ΔΨplasma = 30–60 mV) (Figure 1) [47,58].

**Figure 2:**
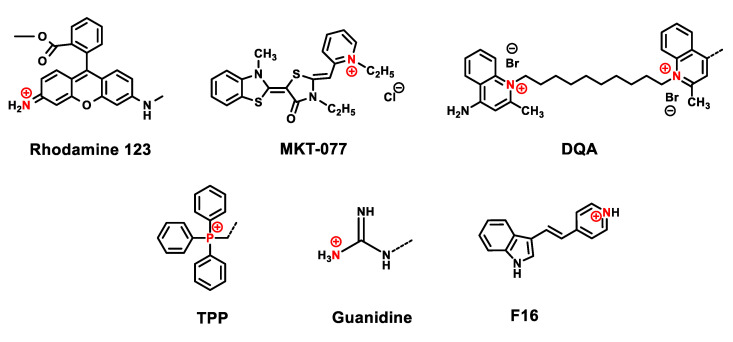
Some common mitochondria-targeting cations.

The simplified passage of the above cationic compounds through the membrane has been thoroughly studied and is accounted for by the large hydrophobic surface and large ionic radius of the cation. Delocalized lipophilic cations that penetrate the hydrophobic barriers of plasma and mitochondrial membranes include Rhodamine-123, rhodacyanine MKT-077, dequalinium, triphenylphosphonium, guanidinium cations and the recently discovered cationic molecule F16 (Figure 2).

Lipophilic cations have different chemical structures and possess different mechanisms of mitochondrial toxicity. For example, dequalinium chloride inhibits NADH-ubiquinone reductase of the mitochondrial respiratory chain [9,59], promoting ROS overproduction and induction of the MPT pore [60]. Rhodamine-123 disrupts the bioenergetic functions of mitochondria by ATP synthase inhibition [61]. F16 compound induces apoptosis by decreasing mitochondrial resistance to the induction of calcium dependent MPT pore [62]. The rhodacyanine analogue MKT-077, also known as FJ-776, exhibited the most favorable pharmacological and toxicological profile of the above listed lipophilic cationic compounds. This compound brought about general destabilization of the mitochondrial membrane and triggered nonspecific inhibition of membrane-bound enzymes [63,64]. Preclinical studies of the mitochondrial toxicity of MKT-077 against the mitochondria of the human colon carcinoma cell line and the mitochondria of normal epithelial cells indicated its acceptable therapeutic index. However, clinical trials of MKT-077 were stopped already at the first stage of the clinical triad due to the recurrent renal toxicity of this compound [65,66].

The therapeutic effect and reduction of side effects observed for the above-described cations at high concentrations can be achieved by involving small lipophilic molecules as delivery systems of compounds with known antitumor activity, demonstrated in preclinical or clinical studies. Initially, this approach was successfully implemented using the well-proven triphenylphosphonium cation in the development of mitochondria-targeted antioxidants blocking accumulation of reactive oxygen species in the cell under oxidative stress. The reliability and versatility of this strategy have been demonstrated both in vitro and in vivo experiments. The most significant contribution to this area of research was made by V.P. Skulachev (decyl-TPP^+^ with plastoquinone, SkQ1) and M.P. Murphy and R.A.J Smith (decyl-TPP^+^ with coenzyme Q10, MitoQ) [67,68,69,70,71].

To date, Visomitin eye drops and ointment for cuts and burns based on SkQ1 have been developed, while the MitoQ compound is undergoing clinical trials as a drug for the treatment of hepatitis C, ischemia-reperfusion syndrome and Parkinson’s disease. Further development of the above strategy relates to possible application of lipophilic carriers for delivery into mitochondria of not only redox-active molecules, but also other biologically active compounds, including toxic small molecules, as targeted antitumor agents. Generally, all of the above cationic small molecules can be successfully employed for selective delivery of cytotoxic substances into the mitochondria of tumor cells. However, among these positively charged lipophilic compounds, only the triphenylphosphonium cation has been thoroughly studied [48,72,73,74,75,76]. Enhanced therapeutic effect has been identified in mitochondria-targeted triphenylphosphonium derivatives of known anticancer drugs such as chlorambucil [73], doxorubicin [74], metformin [75] and tamoxifen [76].

Mito-chlorambucil, tested in breast and pancreatic cancer cell lines, interacted with mtDNA to induce cell death at significantly lower concentrations than the parent chlorambucil compound. Mitochondrial delivery of doxorubicin modified with triphenylphosphonium cation addressed the problem of resistance of human cancer cells MDA-MB-435 to this drug [74]. Impressive result was achieved by TPP^+^ binding to metformin [75] and tamoxifen [76]. Particularly, recent in vitro studies have discovered that metformin, an antidiabetic drug known since the 1950s, has antitumor effect against pancreatic cancer. The study of the mechanism of antitumor action of metformin revealed that this compound primarily targets complex I (CI) of the mitochondrial respiratory chain. The attachment of the TPP^+^ fragment to metformin through the C-10 alkane spacer increased the toxicity of the parent drug by three to four orders of magnitude, proving the triphenylphosphonium metformin derivative Mito-Met to be a promising anticancer agent [75]. Tamoxifen, a mixed estrogen receptor (ER) agonist/antagonist, is a part of first-line therapy for hormone-sensitive breast cancer but is ineffective in the treatment of Her2-dependent tumor type. The conjugate of tamoxifen with the mitochondria-targeted TPP^+^ group has proved highly effective against tumor cells with high levels of Her2 [76]. Inhibition of the mitochondrial complex I and a significant increase in the production of reactive oxygen species in cancer cells account for its cytotoxicity. Mito-Tam and Mito-Met have efficiently completed Phase 1 of clinical trials.

Along with the development of strategies for treatment of oncological diseases with mitochondria-targeted derivatives of known synthetic drugs, there is an active search for mitocans among natural plant substances, referred to as “Herbal mitocans” in the literature [77]. In this case, both medicinal plant extracts and individual molecules are under study. The most striking result among natural mitocan compounds was demonstrated by alpha-tocopherol succinate (α-TOS) and its triphenylphosphonium derivative (MitoVES).

These promising anticancer agents, the first members of the mitocan class, were discovered and systematically studied by Jiri Neuzil et al. [6,7,72,78,79,80]. The study of the mechanism of antitumor action demonstrated that vitamin E group mitocans suppress the activity of complex II of the mitochondrial respiratory chain, preventing the access of coenzyme Q to the respiratory chain complexes. At the same time, these agents initiated enhanced generation of reactive oxygen species, triggering selective apoptosis in cancer cells. Furthermore, MitoVES predominantly binds to the mitochondria of cancer cells and kills cancer cells much more efficiently than the original α-TOS compound. At present, α-TOS and MitoVES have successfully passed the stage of preclinical studies directed at human breast cancer. Inhibition of complex II by vitamin E group mitocans can lead to the accumulation of succinate, which is known to inhibit prolyl hydroxylase and mediate the stabilization of HIF1a and, in turn, causes stimulation of the glycolytic pathway [81]. One could assume that in some cases, this can, on the contrary, facilitate tumorigenesis and progression.

The polyphenolic compound curcumin and its analogue Mito-curcumin are also being investigated as promising drug candidates. Curcumin can suppress proliferation and survival of almost all types of tumor cells. However, its efficiency is limited by low bioavailability in blood plasma and tissues, which is insufficient for intracellular accumulation. In preclinical trials, Mito-curcumin exhibited significant cytotoxicity and antiproliferative activity against MCF-7, MDAMB-231, SKNSH, DU-145 and HeLa cancer cells with a much lower IC_50_ value compared to curcumin [82,83].

## 4. Mitochondria-Targeted Conjugates of Triterpenic Acids with DLCs as a Novel Group of Mitocans

**Figure 3:**
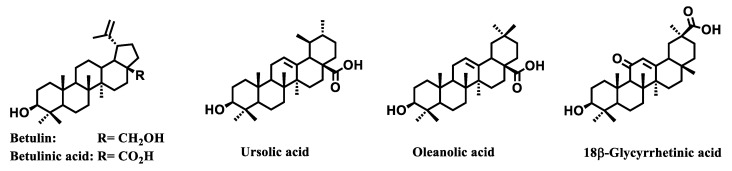
Molecular structures of betulin, betulinic, ursolic, oleanolic and 18β-glycyrrhetinic acids.

Pentacyclic triterpenoids (betulin, betulinic, ursolic, oleanolic and glycyrrhetic acids) are one the most available terpenoids in the plant kingdom (Figure 3).

These compounds are of great interest for pharmacological studies as they exhibit a wide range of biological effects [11,84,85,86,87,88]. These distinguishing properties of triterpenoids include anticancer effect and ability to trigger the mitochondrial pathway of apoptosis in various types of human cancer cells. Indeed, betulinic acid can induce apoptosis in tumor cells of melanoma, lung cancer, ovarian cancer and neuroectodermal tumors [18,89,90]. Betulinic acid stimulates apoptosis involving reactive oxygen species (ROS). ROS promote the permeability of the outer mitochondrial membrane (OMM) by releasing apoptogenic mitochondrial proteins (cytochrome *c*, Smac, apoptosis inducing factor (AIF)) from the intermembrane space, followed by activation of the caspase cascade [18,89,90,91,92]. Ursolic acid has been shown to inhibit the proliferation of various types of cancer cells by suppressing the STAT3 activation pathway [12,93]. Ursolic acid can also induce apoptosis, autophagy and cell cycle arrest in various ways, such as inhibiting DNA replication, stimulating production of reactive oxygen species and influencing the balance between pro- and anti-apoptotic proteins [94,95,96]. Native betulin and betulonic acid were found to directly affect mitochondria and their membranes, inhibiting the activity of complexes of the respiratory chain of organelles, thereby initiating ROS overproduction and mitochondrial dysfunction [97]. The lack of cytotoxic effect against normal human cells (fibroblasts or normal lymphocytes) confers a significant advantage to triterpenic acids. However, a relatively low antitumor potential, high hydrophobicity, poor solubility in blood serum significantly complicate promotion of triterpenoids as anticancer drug candidates.

**Figure 4:**
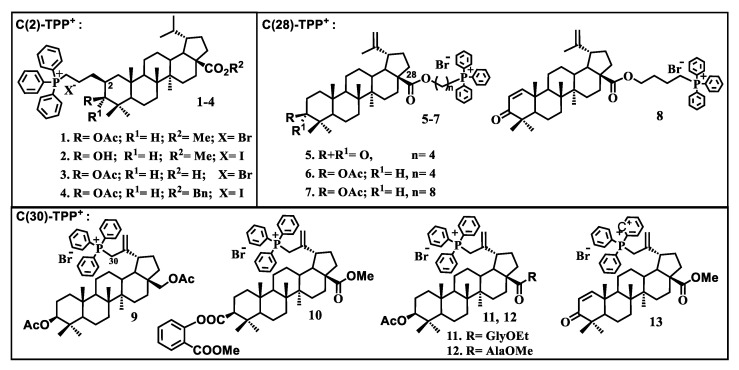
Molecular structures of triphenylphosphonium salts **1**–**13**.

So far, mitochondria-targeted pentacyclic triterpenoids, with the triterpene skeleton bonded to a lipophilic cationic fragment, remain a poorly studied class of compounds [98,99,100,101,102,103]. Our research group synthesized the first samples of triphenylphosphonium derivatives of betulinic acid in 2013 [98] (Figure 4).

**Figure 5:**
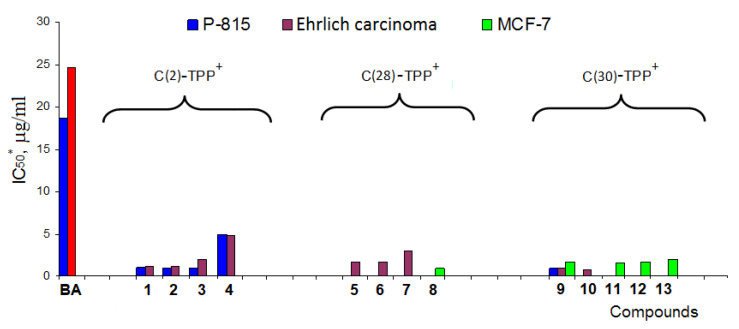
Cytotoxic activity data for betulinic acid (BA) and triphenylphosphonium salts **1**–**13** against P-815, Ehrlich and MCF-7. * IC_50_ is the concentration of compounds that inhibits 50% of cell growth. Adapted from [99].

The addition of a triphenylphosphonium fragment to the betulinic acid molecule at the C-2 position of ring A through the alkyl chain produced a 40–50-fold increase in the cytotoxic effect of cationic derivatives compared to betulinic acid. Subsequent studies on the antitumor activity of a large number of triphenylphosphonium salts of lupane triterpenoids proved that TPP^+^ has a major impact on the cytotoxicity of cationic compounds irrespective of the structure of the triterpene skeleton and the position the cationic TPP^+^ fragment is attached to [99] (Figure 5).

**Figure 6:**
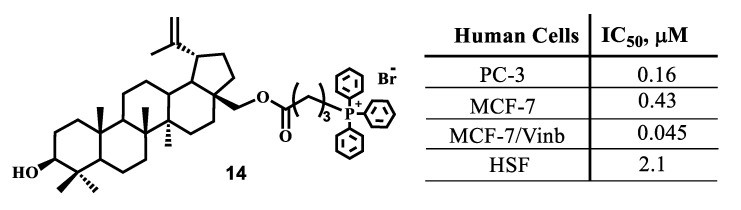
Molecular structure and cytotoxic activity data for triphenylphosphonium salt **14**.

The cytotoxic effect of triphenylphosphonium derivatives of betulin triterpenoid against adenocarcinoma of the prostate (RC-3) and human breast cancer (MCF-7), including the vinblastine-resistant type of cancer MCF-7/Vinb, was analyzed [100]. The findings revealed that the conjugate of betulin with TPP^+^ considerably increased, compared to betulin, the antiproliferative effect against vinblastine-resistant MCF-7 cells with an IC_50_ value of less than 0.045 μM (Figure 6).

**Figure 7:**
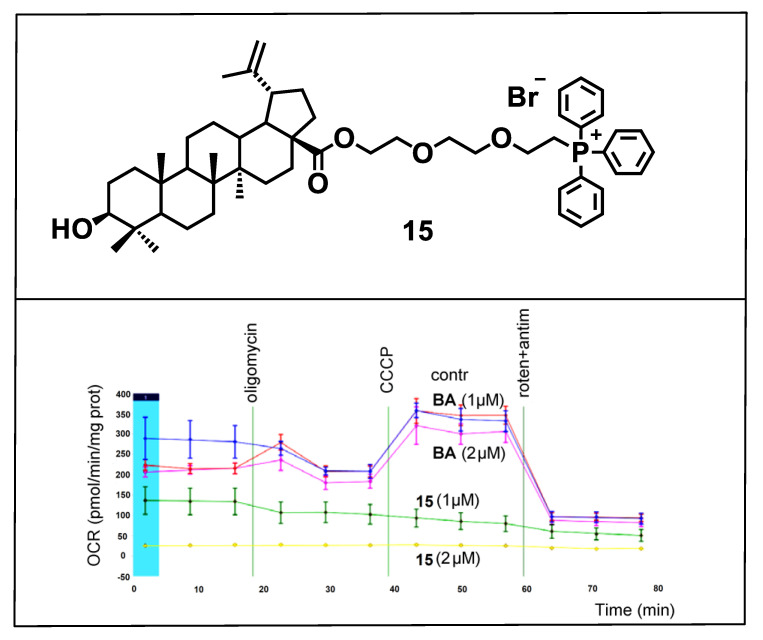
Assessment of oxygen consumption by HCT116 (human colon carcinoma) cancer cells after incubation with betulinic acid **BA** and triphenylphosphonium salt **15**, for 24 h. Adapted from [101].

The studies of the mechanism of cytotoxic effect of triterpenoid-TPP^+^ conjugates carried out in [101,102,103] indicated that triterpene mitocans initiate the mitochondrial pathway of apoptosis in cancer cells, producing reactive oxygen species and decreasing the mitochondrial membrane potential. In addition, assessment of oxygen consumption by HCT116 (human colon carcinoma) tumor cells after incubation with betulinic acid and triphenylphosphonium derivative **15** confirmed that conjugate **15** suppresses mitochondrial respiration at low concentrations (1–2 μM). At these concentrations, betulinic acid was ineffective (Figure 7).

**Figure 8:**
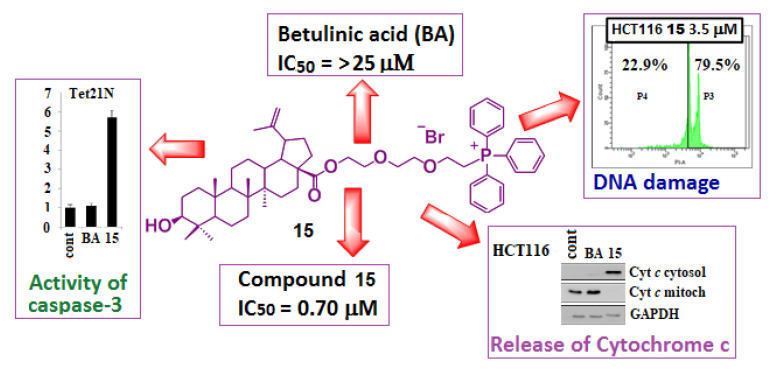
Cytotoxicity and antitumor activity analysis of triphenylphosphonium derivative of betulinic acid. Adapted from [101].

Furthermore, treatment of HCT116 and Tet 21N tumor cells with conjugate 15 brought about disruption of PARP, a significant increase in caspase-3 activity and release of cytochrome *c* into the cytosol (Figure 8). These experiments clearly demonstrate mitochondrial involvement in triggering apoptosis of BA analog 15 [101].

**Figure 9:**
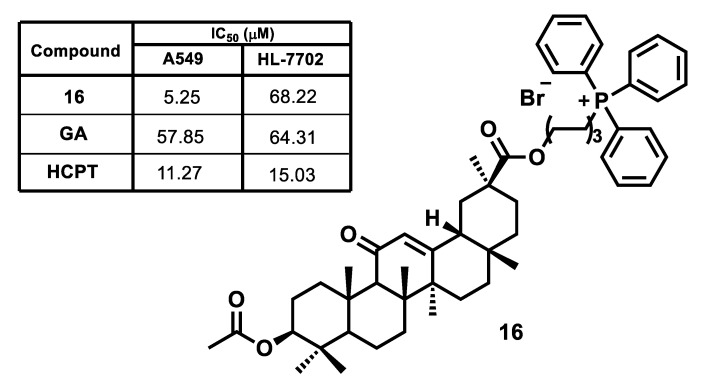
Cytotoxicity of glycyrrhetinic acid GA, triphenylphosphonium salt 16 and 10-hydroxycamptothecin HCPT.

The prospects of applying a mitochondria-targeted strategy for enhancing the cytotoxic effect and selectivity of the antitumor action of triterpenic acids have been convincingly demonstrated in the study of conjugates of glycyrrhetinic acid (GA) with a TPP^+^ fragment [102]. Triphenylphosphonium derivative **16** exhibited significantly higher antitumor activity, as well as acceptable selectivity for the studied cancer and normal cells, compared to GA and 10-hydroxycamptothecin (HCPT) (Figure 9).

Mitochondrial uptake of compound **16** in human lung cancer A549 increased 2.5-fold compared to natural GA. The analysis of the mechanism of the pro-apoptotic action of conjugate **16** by flow cytometry and western blotting methods showed that after incubation of A549 cancer cells with compound **16** (5, 10 and 20 μM), the percentages of early and late apoptosis cell was 18.5%, 30.8% and 45.0%, respectively. Moreover, triphenylphosphonium derivative **16** triggered apoptosis of A549 cells through the inner mitochondrial pathway via reactive oxygen species production, the collapse of mitochondrial membrane potential, the activation of caspases-9 and caspases-3. Conjugate **16** significantly increased the expression of pro-apoptotic protein Bax and corresponding down-regulated the expression of anti-apoptotic protein Bcl-2.

The ability to induce a mitochondria-dependent pathway of apoptosis in cancer cells of various etiologies by the action of triphenylphosphonium derivatives of lupane triterpenoids was reported by Fan P. et al. as well [103]. More importantly, mitochondria-targeted triterpenoid derivatives significantly inhibited cancer cell proliferation and migration in an in vivo zebrafish xenograft model.

Recently, the mitochondria-toxic cationic compound F16 was found to selectively accumulate in the mitochondrial matrix of various tumor cell lines [57,62]. Its high concentration in mitochondria leads to cell death caused by the arrest of the cell cycle, interruption of the mitochondrial respiratory chain, a decreased intracellular ATP level and induction of apoptosis. Meanwhile, the potential of F16 for delivering biologically active compounds to malignant transformed cells, unlike the triphenylphosphonium cation typically used today, has not been studied in detail and is reported only in a few works [104,105]. To reduce side effects, the known antitumor agent 5-fluorouracil (5-FU) was linked to F16 via ester, amide, or sulfide functions in the work [104]. The resulting conjugates did not show the expected synergistic effect though, presumably on account of different mechanism of antitumor activity of fluoronucleotide 5-FU, which disrupts the sequence of RNA and DNA chains and F16, concentrated in the mitochondrial matrix.

Instead, the conjugate of the antitumor alkylating agent chlorambucil with F16 offered good prospects for further research as a drug candidate [105]. This hybrid compound accumulated mainly in the mitochondria of cancer cells and simultaneously affected several mitochondrial components, interacted with mtDNA, increased the concentration of reactive oxygen species and caused depolarization of the inner mitochondrial membrane. The above examples suggest that the molecular structures of the antitumor compound and the cationic fragment used as a carrier can have a significant impact on cytotoxicity, cellular penetration and the mechanism of the antitumor action of the target conjugates. We assumed that the combination of the apoptosis-inducing triterpenoid molecule with the cationic F16 fragment would enhance the antitumor effect of the hybrid compound, similar to the use of TPP^+^ cation.

**Figure 10:**
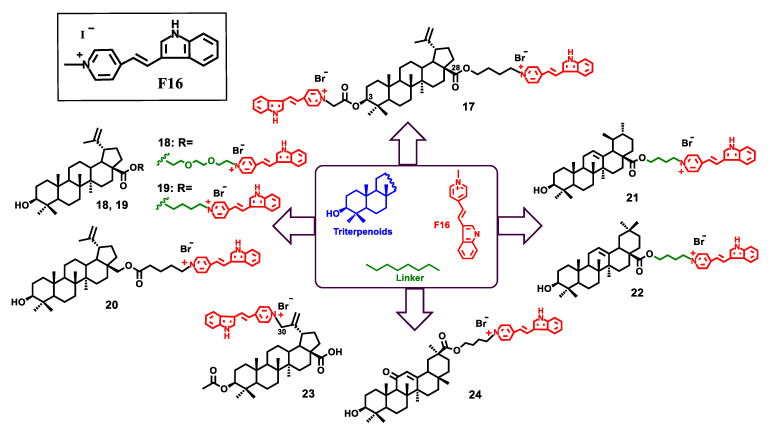
Molecular structures of F16 and new conjugates of triterpenoids **17–24**.

The synthesis of conjugates of F16 with betulin, betulinic, ursolic, oleanolic and glycyrrhetic acids was carried out by binding the triterpene nucleus at the C-3, C-28 or C-30 positions with one or two F16 fragments, via butane or triethylene glycol spacers [106] (Figure 10).

The resulting conjugates 17–24, betulinic acid and compound F16, as well as a mechanical equimolar mixture of betulinic acid and F16 (1:1, mol/mol) were tested in vitro in three tumor cell lines U937 (leukemic monocytic lymphoma), K562 (chronic myeloid leukemia), Jurkat (T-lymphoblastic leukemia) and a healthy human fibroblast cell line. Cell viability after exposure to the test compounds was analyzed by flow cytometry.

Most hybrids 18–20, 21, 22 and 24 exhibited high cytotoxic activity against all cancer cell lines under study. These compounds were considerably (≈100–200 times) more cytotoxic than the parent betulinic acid (Table 1).

Lupane triterpenoids **18**–**20** were found to be the most selective compounds among the studied conjugates with a selectivity index of about 10 (determined by the IC_50_ ratio of the U937 tumor cell line to non-malignant fibroblasts). The introduction of the second fragment (E)-4-(1H-indole-3-ylvinyl) pyridine into the triterpenoid molecule (compound **17**, IC_50_, 4.19 μM, U937), as well as conjugation of betulinic acid with F16 at the position C-30 did not increase the antitumor activity (compound **23**, IC_50_, >125 μM). Compound F16 did not show cytotoxic action under studied conditions, while betulinic acid BA exhibited antitumor activity with IC_50_ values of 149 (U-937), 81.7 (Jurkat) and 78.5 (K562) mM. Compared to the covalent binding of betulinic acid to the **F16** molecule, the mechanical mixture of these compounds did not show a noticeable increase in the cytotoxic effect though (Table 1).

**Figure 11:**
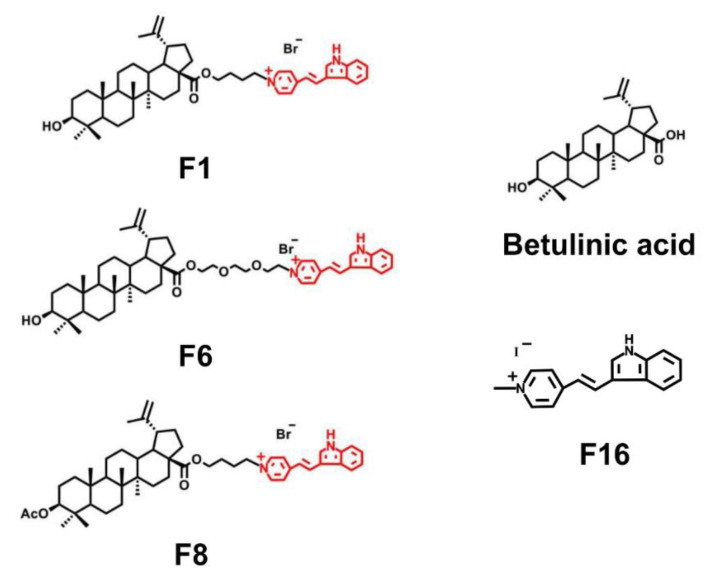
Structure of conjugates and **F16**-betulinic acid (**F1**, **F6**, **F8**).

Recently, we reported the direct effects of new F16—betulinic acid [107] and F16—betulin conjugates [108] on mitochondria. The conjugates were shown to be capable of targeted binding to mitochondrial membranes and dose-dependently reducing the membrane potential of organelles, as well as the intensity of respiration and oxidative phosphorylation, which is also accompanied by an increase in the production of hydrogen peroxide by mitochondria (Figure 11).

The membranotropic activity of F16—betulinic acid and F16—betulin conjugates was found to be the result of several effects: reversion of organelle ATP synthase, inhibition of the activity of the respiratory chain complexes and, first of all, complex I, which is the main generator of ROS in mitochondria. Moreover, it was found that the F6 conjugate, due to its structure (the presence of oxygen atoms in the triethylene glycol spacer connecting betulinic acid and F16) proved to induce permeabilization of the lipid phase of the inner mitochondrial membrane, as well as the membrane of liposomes. In this case, the F6 conjugate is supposed to serve a protonophore uncoupler and transfer protons across the inner mitochondrial membrane. We also demonstrated the mitochondria-targeted effects of conjugates on rat thymocytes cells. Indeed, all the studied conjugates were able to reduce the mitochondrial potential of these cells, as well as initiate superoxide overproduction, which probably provides their cytotoxic effect. The conjugates are considered to have a similar effect on the mitochondria of cancer cells. Meanwhile, the previously revealed selectivity of agents allows to expect more pronounced mitochondria-targeted cytotoxic effects on cancer cells with a high mitochondrial membrane potential. Our research group is planning to address this important issue in further studies.

**Figure 12:**
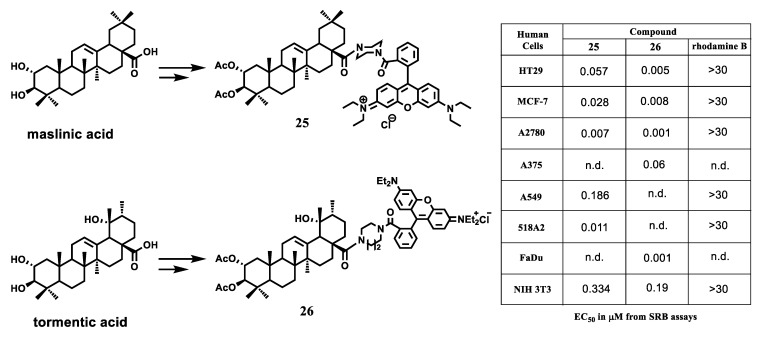
Cytotoxicity of maslinic-RhoB and tormentic-RhoB conjugates. Adapted from [109,115].

Recently R. Csuk et al. reported the development of triterpenoid rhodamin B conjugates as promising new mitocans exhibiting cytotoxicity against various human cell lines at low nanomolar concentrations [109,110,111,112,113,114,115,116]. A significant increase in the cytotoxic effect of natural triterpenic acids (for example, ursolic, oleanolic, betulinic, maslinic acid) was achieved by their combination with rhodamin B (RhoB) through a piperazine spacer. Moreover, the resulting hybrid compounds showed low toxicity to healthy mouse fibroblast cells. Thus, the conjugate of 2,3-di-O-acetyl maslinic acid and RhoB 25 was approximately 1000 times more cytotoxic and far more selective (Fsi = 5 0, defined as EC_50_ A2780 tumor cell line to EC_50_ nonmalignant mouse fibroblasts NIH 3T3) than its prototype maslinic acid [109] (Figure 12).

The antitumor activity of hybrid **25** was comparable to common anticancer drugs such as doxorubicin and paclitaxel. RhoB showed no cytotoxicity up to a concentration of 30 μM (experimental threshold value). The morphological changes in A2780 (ovarian carcinoma) cancer cells after incubation with compound 25 were analyzed with a fluorescence microscope and double staining experiments. The research outcomes proved conjugate 25 to function as a mitocan agent.

The study of the mechanism of mitochondrial action of triterpenoid-RhoB conjugates applying a molecular docking approach indicated that these hybrid compounds can target mitochondrial NADH dehydrogenase (complex I) and mitochondrial succinate dehydrogenase (complex II), enzymes responsible for the transfer of electrons in the mitochondrial respiratory chain and formation of reactive oxygen species in mitochondria [117]. Further studies included synthesis of a broad range of rhodamine derivatives of triterpenic acids [110,111,112,113,114,115,116] and RhoB conjugates with some steroids and diterpenoids. The RhoB fragment was attached to the triterpene nucleus directly as a triterpene ester, or the triterpenoid and lipophilic cation fragments were separated by piperazine or homopiperazine spacers. The analysis of cytotoxic activity of the conjugates identified the crucial importance of a triterpene nucleus in the hybrid compound, while the molecular structure of the terpene skeleton and the ring size of the heterocyclic spacer (piperazine, homopiperazine) had a significant impact on the biological effect of the hybrid. The homopiperazine spacer was clearly superior to the piperazine fragment. The authors consider the tormentic acid (TA) and RhoB conjugate with a homopiperazine spacer 26 to be the most promising candidate for further biological studies among the compounds studied [115].

## 5. Conclusions

In the past few years, secondary plant metabolites, pentacyclic triterpenic acids, have been actively studied as a promising molecular platform for development of a libraries of antitumor mitochondria-targeted agents inducing various forms of cell death. These natural compounds are distinguished by their availability and cytotoxic activity, established against various types of tumor cells. The antitumor effect of pentacyclic triterpenic acids is directed at different cellular targets, but their cytotoxic activity for the most part is associated with affecting the mitochondria of cancer cells. Triterpenic acids directly affect mitochondrial membranes and, first of all, the respiratory chain, initiating overproduction of reactive oxygen species in these organelles, resulting in the induction of mitochondrial permeability transition (MPT), release of cytochrome *c* into the cytosol and finally induction of cell death.

Furthermore, these natural compounds inhibit cancer cell proliferation through cell cycle arrest and suppress tumor angiogenesis by blocking epidermal growth factor receptors and nuclear transcription factor NF-kB, which affects various aspects of angiogenesis. However, the low bioavailability of triterpenoids does not allow them to reach the target in vivo and obtain the desired therapeutic effect in acceptable therapeutic doses. The research results highlighted in this article prove the effectiveness of natural triterpenic acids to be improved by binding to mitochondria-targeted carrier molecules. And although the mechanisms of action of mitocans based on delocalized cations and triterpenes on mitochondria may be specific, one way or another, they all cause generalized mitochondrial dysfunction with varying efficiency. Therefore, an important task is to carefully select the configuration and concentration of mitocan for selective action on tumor cells.

At the same time, to date, none of the antineoplastic agents mitocans has been introduced to the pharmaceutical market. This is primarily due to the fact that most of the data was obtained in vitro on cancer cells or, in one case, on the zebrafish xenograft model. It is necessary to thoroughly test the efficacy of TPP-conjugated triterpenes in vivo, using advanced mouse models that recapitulate landmark events in human tumors. This will show whether these mitocans are well tolerated for prolonged times, without toxicity in the organs with prevalent oxidative metabolism (heart, brain, muscles). In addition, it is necessary to assess their effect at different stages of the disease, given that metabolic phenotypes develop with tumor progression and late stages of the disease show an increase in the dependence of tumor cells on OXPHOS. Perhaps in this case mitocans causing generalized mitochondrial dysfunction will be useful. This implies not only the creation of a special animal model, reproducing human tumors cancer progression, but also the identification of biomarkers in order to predict the patients who would benefit from them and the best therapeutic time window.

It is also important to note that targeting mitochondria cannot be considered a general anti-cancer approach but is likely to be effective in those tumors that have been shown to be highly dependent on oxidative metabolism, such as brain cancer, acute myeloid leukemia [39,118], cisplatin-resistant ovarian cancer cells [119] or at certain stages of tumor progression [22].

## Figures and Tables

**Table 1:** Cytotoxic action of compounds 17–24 on the U937, Jurkat and K562 tumor cells and fibroblasts (X ± SE) *^b^*.

Compound	IC_50_ (μM) *^a^*			
	U937	Jurkat	K562	Fibroblasts
**17**	4.190 ± 0.117 *^b^*	4.360 ± 0.122 *^b^*	4.010 ± 0.109 *^b^*	10.400 ± 1.230 *^b^*
**18**	0.573 ± 0.024 *^b^*	1.260 ± 0.042 *^b^*	1.210 ± 0.041 *^b^*	5.500 ± 0.340 *^b^*
**19**	0.616 ± 0.028 *^b^*	0.844 ± 0.034 *^b^*	0.812 ± 0.032 *^b^*	6.100 ± 0.220 *^b^*
**20**	0.906 ± 0.037 *^b^*	0.937 ± 0.032 *^b^*	0.904 ± 0.033 *^b^*	8.200 ± 0.630 *^b^*
**21**	2.461 ± 0.085 *^b^*	0.623 ± 0.031 *^b^*	0.588 ± 0.032 *^b^*	6.230 ± 0.850 *^b^*
**22**	0.607 ± 0.027 *^b^*	0.687 ± 0.034 *^b^*	0.671 ± 0.035 *^b^*	3.490 ± 0.560 *^b^*
**23**	>125 *^b^*	>125 *^b^*	>125 *^b^*	>125 *^b^*
**24**	2.425 ± 0.083 *^b^*	0.559 ± 0.024 *^b^*	0.511 ± 0.022 *^b^*	8.300 ± 1.190 *^b^*
**F16**	>500 *^b^*	>500 *^b^*	>500 *^b^*	>500 *^b^*
**BA** *^c^*	149.290 ± 4.170 *^b^*	81.680 ± 1.820 *^b^*	78.540 ± 1.760 *^b^*	236.400 ± 3.600 *^b^*
**F16:BA/1:1**	122.170 ± 3.460 *^b^*	91.580 ± 1.950 *^b^*	89.150 ± 1.890 *^b^*	280.100 ± 3.440 *^b^*

*^a^* IC_50_ (μM) is the half-maximal inhibitory concentration against the tested cells *^b^* X is the average of experimental values, SE is the standard error. Each IC_50_ value (X ± SE) was found from the data of three experiments performed in duplicate. *^c^* BA is the betulinic acid. Adapted from [106].

## Data Availability

The data presented in this study are available on request from the corresponding author.
